# Transforming Obesity Care With Digital Therapeutics: Mobile Health Apps, Clinical Evidence, and Regulatory Considerations

**DOI:** 10.1155/jobe/9482093

**Published:** 2026-10-19

**Authors:** Mayuri, Bidisha Bhattacharya, Kunal Kar, Pravin Pawar, Inderbir Singh, Tanikan Sangnim, Kampanart Huanbutta

**Affiliations:** ^1^ Chitkara College of Pharmacy Chitkara University Rajpura Punjab India chitkara.edu.in; ^2^ Dr. Shivajirao Kadam College of Pharmacy Sangli Maharashtra India; ^3^ Faculty of Pharmaceutical Sciences Burapha University Chonburi 20131 Thailand buu.ac.th; ^4^ Department of Manufacturing Pharmacy College of Pharmacy Rangsit University Pathum Thani 12000 Thailand rsu.ac.th

**Keywords:** artificial intelligence, digital therapeutics, mobile applications, obesity management, software as a medical device

## Abstract

Obesity remains a complex global public health crisis driven by environmental, biochemical, and behavioral factors, leading to severe metabolic comorbidities. Digital therapeutics (DTx) has emerged as a scalable, data‐driven intervention, utilizing wearable sensors, mobile applications, and artificial intelligence (AI) to deliver personalized weight‐management strategies. Rather than relying solely on traditional approaches, true DTx distinguishes it through rigorous clinical validation and targeted behavioral modification. Current evidence from clinical trials and real‐world data indicates that while DTx effectively enhances patient engagement and promotes short‐term behavioral change, sustaining long‐term adherence and significant weight loss remains a critical challenge. Furthermore, the integration of AI models offers advanced personalization, though their efficacy must be balanced against potential algorithmic biases and traditional statistical approaches. Successful implementation of DTx into standard obesity care requires overcoming substantial barriers, particularly digital divide, data privacy concerns, and fragmented regulatory frameworks. Ultimately, realizing the full potential of DTx necessitates collaborative efforts to establish standardized regulations, ensure equitable access, and design evidence‐based interventions that prioritize long‐term patient engagement and clinical efficacy.

## 1. Obesity

### 1.1. Overview and Clinical Definition

Obesity is a complex, chronic medical condition characterized by an abnormal or excessive accumulation of adipose tissue, primarily due to an increase in both the size and number of fat cells, which poses significant health risks. It arises from a multifactorial interplay of biological, environmental, and lifestyle‐related factors. Globally, obesity affects approximately 650 million individuals, making it a major public health concern [1, 2]. The World Health Organization (WHO) defines obesity using the Body Mass Index (BMI): a BMI of 25.0–29.9 kg/m^2^ indicates overweight and ≥ 30.0 indicates obesity, further stratified into Class 1 (BMI 30.0–34.9), Class 2 (BMI 35.0–39.9), and Class 3 (BMI ≥ 40.0). These international cutoff points are not, however, applied uniformly across populations. For Asian populations, the Asia‐Pacific guidelines propose lower BMI thresholds, with a BMI of 23.0–24.9 kg/m^2^ classified as overweight and a BMI ≥ 25.0 kg/m^2^ classified as obesity, reflecting evidence that Asian populations may experience increased cardiometabolic risk at lower BMI levels than European populations. The WHO Expert Consultation similarly recognized that the risk of type 2 diabetes and cardiovascular disease increases at lower BMI levels in Asian populations, although it retained the existing WHO international BMI classifications and identified 23.0 and 27.5 kg/m^2^ as additional public‐health action points. Throughout this review, the criterion applied is stated explicitly wherever prevalence data are reported, because estimates derived from the WHO international criterion and the Asia‐Pacific criterion are not directly comparable [3].

Obesity significantly increases the risk of developing a range of metabolic and systemic complications, including type 2 diabetes, metabolic dysfunction‐associated steatotic liver disease (MASLD), cardiovascular diseases, musculoskeletal disorders such as joint pain, and respiratory conditions like obstructive sleep apnea (OSA) [4, 5]. Furthermore, obesity impacts the immune system by reducing the ability of T‐cells and B‐cells to grow and multiply. Macrophage aggregates have been observed to grow larger as obesity severity increases, suggesting they might be utilized to gauge the inflammatory condition associated with obesity [6]. Excess fat accumulation in the abdominal region, known as “abdominal obesity,” further exacerbates these health risks, although its measurement guidelines differ from those of general obesity [7].

Lifestyle interventions, including dietary modification, increased physical activity, and behavioral therapy, remain central to the management and prevention of obesity [8, 9]. However, one of the major challenges in long‐term management is weight regain following initial weight loss. This phenomenon is primarily attributed to a persistently reduced resting metabolic rate and an increase in appetite, driven by enduring changes in appetite‐regulating pathways, characterized by sustained elevations in orexigenic (hunger‐promoting) signals and reductions in anorexigenic (satiety‐promoting) signals [10].

Given these clinical challenges, digital health technologies have emerged as promising tools to support sustainable weight management. This review was conducted to identify and evaluate the available evidence on the role of DTx in obesity management, with particular emphasis on mobile health applications, clinical outcomes, and regulatory considerations.

### 1.2. Determinants and Etiology of Obesity

To meet energy demands, the human body metabolizes carbohydrates, proteins, and fats. During periods of food scarcity, fat serves as the primary energy reserve [11]. While heredity plays a role in energy balance, weight management, and fat storage, it alone is insufficient to explain the rapidly rising obesity rates in countries such as the United States and the United Kingdom. Instead, the obesity epidemic is largely driven by a gene–environment interaction, wherein genetically predisposed individuals are exposed to an environment characterized by energy‐dense diets and sedentary lifestyles [12, 13].

As a complex disorder, obesity is influenced by a multitude of biological, behavioral, social, and environmental variables. While genetics, metabolism, and food tolerance constitute biological factors, individual behaviors such as dietary habits and physical activity profoundly impact body weight. A chronic positive energy balance, resulting from the overconsumption of energy‐dense foods and physical inactivity, ultimately leads to weight gain [14]. The prevalence of obesity is further impacted by social determinants, including socioeconomic status, cultural background, and education level. For instance, lower‐income populations frequently face reduced access to safe recreational spaces and nutritious food options. An obesogenic environment is further fostered by environmental variables such as the pervasive availability of unhealthy fast food, aggressive marketing of high‐calorie meals, and limited opportunities for physical activity. Therefore, combating obesity and advancing long‐term health solutions necessitate a comprehensive, system‐wide approach that integrates behavioral, social, genetic, and environmental factors [15].

### 1.3. Molecular Pathophysiology and Metabolic Dysfunction

The pathophysiology of obesity, illustrated in Figure 1, involves complex interactions among the brain, adipose tissue, the pancreas, the gut microbiota, and the hormones that regulate metabolism and satiety; the hypothalamus is central to energy homeostasis, and its disruption impairs appetite control and promotes obesity [16].

**FIGURE 1:**
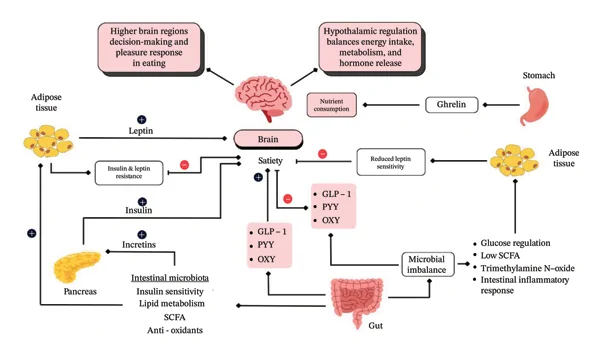
Complex mechanisms underlying obesity pathophysiology and metabolic dysfunction.

Adipose tissue secretes leptin to signal satiety, but obesity frequently induces leptin resistance, leading to continued hunger, reduced energy expenditure, and excess fat storage [17]. Concurrently, blunted post‐prandial ghrelin suppression, pancreatic insulin resistance, and impaired incretin signaling further promote overeating, hinder glucose metabolism, and exacerbate weight gain [7].

Gut microbiota also significantly influences metabolic health by affecting short‐chain fatty acid (SCFA) generation, lipid metabolism, and insulin sensitivity. In obesity, microbial dysbiosis contributes to metabolic dysfunction by interfering with glucose regulation, lowering SCFA levels, increasing cardiovascular‐associated trimethylamine‐N‐oxide (TMAO), and inducing intestinal inflammation. Furthermore, dysbiosis impairs gut hormones that control satiety, such as glucagon‐like peptide‐1 (GLP‐1), peptide YY (PYY), and oxyntomodulin (OXY). The interplay between insulin resistance, gut dysbiosis, hormonal imbalances, and hypothalamic dysregulation creates a vicious cycle that sustains obesity [18].

Digital therapeutics (DTx) offers a promising approach by utilizing behavior modification techniques, real‐time monitoring, and structured lifestyle interventions. While DTx does not directly alter hormones pharmacologically, they indirectly improve insulin sensitivity, support gut health, and enhance satiety signaling by promoting healthy eating habits, physical activity, sleep regulation, and stress management. By targeting the underlying behavioral drivers, DTx helps disrupt the cycle of weight gain and restore metabolic homeostasis [19].

### 1.4. Systemic Comorbidities Linked to Obesity

As illustrated in Figure 2, obesity is a systemic risk factor affecting neurological, cardiovascular, endocrine, respiratory, digestive, musculoskeletal, and reproductive health, including conditions such as idiopathic intracranial hypertension and stroke [20, 21]. It also raises the risk of cardiovascular disease, including coronary heart disease and hypertension, and, through visceral adiposity, insulin resistance [22, 23]. Obesity additionally increases the risk of several malignancies (e.g., colon, pancreatic, breast, and prostate cancers) through hormonal imbalance, metabolic dysfunction, and chronic inflammation [24].

**FIGURE 2:**
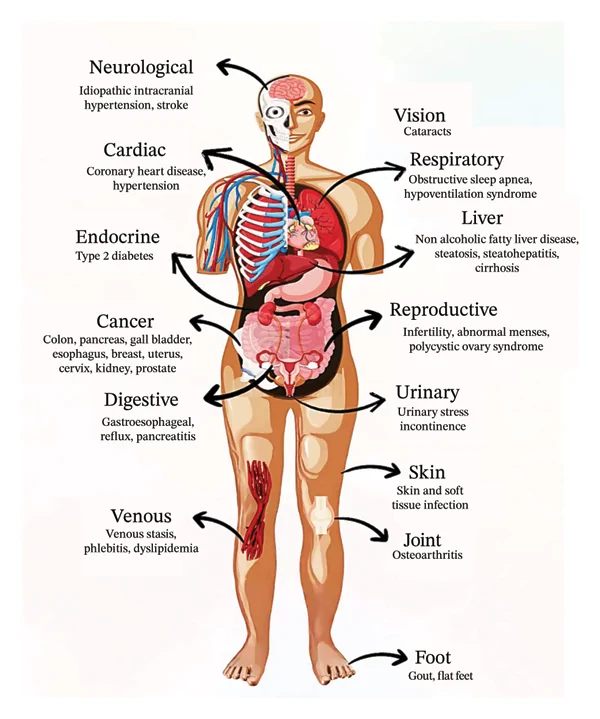
Obesity‐related comorbidities affecting multiple organ systems.

Obesity further contributes to digestive disorders such as gastroesophageal reflux disease and pancreatitis, and to reproductive complications including infertility and polycystic ovary syndrome (PCOS) [25].

Dermatological infections within skin folds and musculoskeletal conditions such as osteoarthritis and gout are also common. Mitigating these comorbidities requires addressing the underlying condition through lifestyle, medical, and public‐health measures [26].

### 1.5. Global and Regional Prevalence: India and Thailand

Overweight and obesity have become major public health concerns in India, with their prevalence increasing substantially over the past 15 years. According to the National Family Health Survey (NFHS‐5; 2019–2021), approximately 24.0% of women and 22.9% of men aged 15–49 years were classified as overweight or obese (BMI ≥ 25.0 kg/m^2^). When obesity was considered separately using the WHO international criterion (BMI ≥ 30.0 kg/m^2^), the prevalence was 6.4% among women and 4.0% among men, representing more than a twofold increase compared with estimates reported in 2005 [27]. In contrast, a separate nationwide analysis applying Asian BMI criteria, which use lower BMI cutoff points, reported an overall obesity prevalence of 40.3%. This substantially higher estimate highlights the importance of clearly specifying the BMI classification criteria used when comparing prevalence across studies, as estimates based on WHO international and Asian BMI criteria are not directly comparable [28].

The burden of overweight and obesity also varies considerably across demographic and socioeconomic groups. Higher prevalence has been reported in southern India, among older adults, particularly those aged 40–49 years, and among individuals in higher socioeconomic groups. However, recent evidence indicates that overweight and obesity are also increasing rapidly among socioeconomically disadvantaged and less‐educated populations, suggesting a narrowing of traditional socioeconomic disparities. The increasing prevalence of overweight and obesity is associated with substantial health consequences, including type 2 diabetes, coronary heart disease, and several cancers, including kidney cancer, thereby underscoring the need for targeted public health interventions and state‐specific prevention strategies [28–30]. The prevalence of overweight and obesity across various Indian states is summarized in Table 1.

**TABLE 1:** State‐wise prevalence of overweight and obesity combined (BMI ≥ 25.0 kg/m^2^, WHO international classification) among men and women aged 15–49 years in India [28].

States	Males (%)	Females (%)
Kerala	24.3	34
Tamil Nadu	19.8	24.4
Andhra Pradesh	17.6	22.7
Punjab	30.3	37.5
Himachal Pradesh	16	19.5
Sikkim	17.3	21
Maharashtra	15.9	18.1
Gujarat	15.4	17.7

In Thailand, obesity has similarly emerged as a significant public health concern. Thailand has been identified as one of the Southeast Asian countries with the fastest‐growing obesity rates between 1995 and 2016, alongside Indonesia, Laos, and Vietnam [31]. It should be noted that Thai national surveys apply the Asia‐Pacific criteria, under which overweight is defined as a BMI of 23.0–24.9 kg/m^2^ and obesity as a BMI ≥ 25.0 kg/m^2^. These estimates should therefore be interpreted according to the BMI classification system used and should not be directly compared with prevalence estimates derived using the WHO international criterion. In the Fourth National Health Examination Survey (NHES IV, 2009), the age‐adjusted prevalence of overweight (BMI 23.0–24.9 kg/m^2^), obesity (BMI 25.0–29.9 kg/m^2^), and severe obesity (BMI ≥ 30.0 kg/m^2^) under the Asia‐Pacific criteria among Thai adults aged ≥ 20 years was 17.5%, 26.0%, and 9.0%, respectively, with higher rates in women and a rising trend over time [32]. This sharp increase is primarily attributed to greater accessibility to energy‐dense, nutrient‐poor foods and a marked transition toward sedentary lifestyles driven by economic development and urbanization [33]. More recent data corroborate this trend; a cross‐sectional study among Royal Thai Army personnel demonstrated a continued rise in obesity prevalence (BMI ≥ 25.0 kg/m^2^, Asia‐Pacific criterion) from 42.1% in 2017 to 44.2% in 2021, particularly among younger personnel aged 18–24 years [34]. Although this occupational cohort is predominantly male and of limited age range and is therefore not representative of the general Thai population, the trend underscores the need for targeted preventive health strategies.

## 2. Methodology and Search Strategy

### 2.1. Study Design and Search Strategy

This narrative review was conducted following the principles of the Scale for the Assessment of Narrative Review Articles (SANRA) guideline to ensure transparency and rigor in literature selection and synthesis. Relevant published studies addressing the effectiveness, technological features, and therapeutic potential of digital interventions for obesity care were gathered through a systematic search of electronic databases, including PubMed, Scopus, Web of Science, and Google Scholar. The search strategy combined keywords and Boolean operators such as: “digital therapeutics,” “mobile health applications,” “obesity management,” “weight loss apps,” “mHealth,” “AI in obesity,” and “behavioral interventions.” In addition, the reference lists of selected articles were manually screened to identify further relevant studies.

### 2.2. Inclusion and Exclusion Criteria

Studies were included if they focused on DTx or mobile health applications specifically targeting obesity or weight management, reported clinical outcomes or behavioral interventions, and were published as peer‐reviewed articles in English involving human participants. Clinical trials, observational studies, and systematic reviews were considered for this review.

Conversely, studies were excluded if they were unrelated to obesity management, described general wellness applications without evidence‐based therapeutic components, or lacked sufficient methodological or outcome data. Furthermore, non‐peer‐reviewed literature, such as editorials, commentaries, or conference abstracts, was excluded to ensure the academic rigor of the synthesis.

### 2.3. Use of Artificial Intelligence (AI) Tools

During the preparation of this review manuscript, the authors used Claude (Anthropic, Claude Opus 4.7) and Gemini (Google, Gemini 3.1 Pro) solely for English language editing, including grammar correction, sentence refinement, and improvement of clarity and readability. These tools were applied only to selected sections of the manuscript. No scientific content, literature interpretation, data analysis, or conclusions were generated by these AI systems. All AI‐assisted outputs were critically reviewed, verified, and edited by the authors, who take full responsibility for the accuracy, integrity, and originality of the final manuscript. The AI tools were not used to generate references, interpret findings, or formulate any recommendations, and they are not listed as authors of this work, in accordance with Wiley’s policy on AI generated content (AIGC).

## 3. DTx

### 3.1. Introduction to DTx and Software as a Medical Device (SaMD)

DTx can be classified under SaMD. They are evidence‐based software interventions developed and designed to prevent, diagnose, cure, and treat disorders. They are a subset of digital health, distinct from general health wellness apps, and require clinical validation and rigorous regulatory oversight [35]. DTx represents a transformative approach in the healthcare scenario, utilizing technology to deliver therapeutic interventions based on evidence rigorously validated through clinical evaluations. These are types of software designed to prevent, diagnose, cure, or manage various diseases, including chronic conditions like type 2 diabetes and mental health disorders.

Unlike general wellness apps, DTx undergoes strict regulatory processes to ensure they meet safety and efficacy standards, requiring approval from regulatory bodies such as the U.S. Food and Drug Administration (FDA), the European Medicines Agency (EMA), and the Central Drugs Standard Control Organization (CDSCO). For instance, DTx can use real‐time data from apps connected to customized wearable devices, providing insights into the patient’s daily activities and health conditions. Moreover, DTx can be employed as standalone software or integrated with traditional pharmacological treatments, providing a holistic approach to healthcare that addresses both physical and mental health needs, ultimately reducing healthcare costs and improving patient engagement [36, 37].

### 3.2. Interrelationship: Digital Health, Digital Medicine, and DTx

Digital health, digital medicine, and DTx represent an emerging landscape in healthcare, driven by the integration of technology to improve patient care and clinical outcomes. Digital health encompasses a broad spectrum of technologies, including telemedicine, mobile applications, and wearable devices, designed to enhance patient connectivity and healthcare accessibility. Digital medicine specifically focuses on evidence‐based software or hardware tools that measure or intervene in human health. These tools often support clinical trials through advanced data analytics and facilitate the diagnosis, treatment, mitigation, and prevention of diseases, emphasizing the importance of randomized clinical trial (RCT) to improve healthcare quality.

The most stringent category is DTx, which delivers evidence‐based therapeutic interventions driven by high‐quality software programs to prevent, manage, or treat a medical disorder or disease. Unlike general wellness applications (e.g., basic calorie trackers), DTx products undergo rigorous clinical evaluation and regulatory oversight to ensure both efficacy and safety. Figure 3 illustrates the interconnected roles of digital health, digital medicine, and DTx in simplifying healthcare delivery and comprehensively addressing patient needs [38].

**FIGURE 3:**
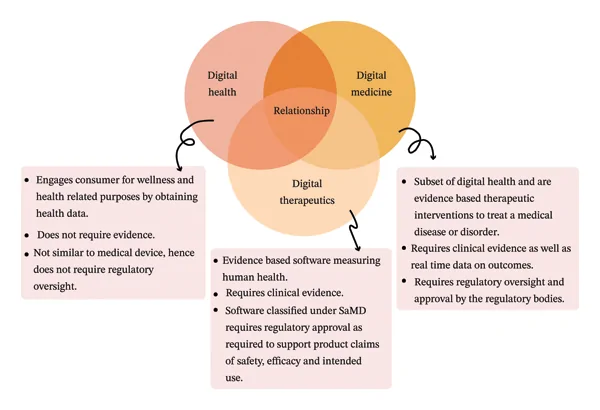
Interconnected roles of digital health, digital medicine, and digital therapeutics.

### 3.3. Mobile Applications and DTx for Obesity Management

DTx applications are emerging as critical tools for facilitating the lifestyle modifications necessary for effective obesity management. These applications utilize evidence‐based interventions delivered via sophisticated software to help individuals improve their health behaviors. Clinical evidence suggests that individuals utilizing DTx platforms for weight loss often achieve significantly better outcomes compared to those relying solely on traditional methods. Furthermore, these applications enhance user engagement through personalized coaching, real‐time data tracking, and behavioral insights [19]. Table 2 presents a selection of mobile applications commonly utilized for weight monitoring and obesity management.

**TABLE 2:** Selection of mobile applications and digital platforms used for obesity management.

Logo	App name	Company/developer	Features	Ref.
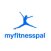	MyFitnessPal	Mike Lee	Calorie tracking, exercise logging, integration with fitness devices, and large food database	[39–42]

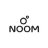	Noom	Saeju Jeong	Behavioral change coaching, personalized plans, calorie tracking, and psychological insights	[43–45]

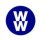	WW (Weight Watchers)	Jean Nidetch	Points‐based food tracking, recipes, personalized coaching, and community support	[46, 47]

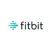	Fitbit	James Park	Activity tracking, calorie burn monitoring, sleep analysis, and personalized health recommendations	[48–50]

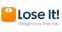	Lose It!	Charles Teague	Food logging, barcode scanning, goal setting, and integration with fitness trackers	[51, 52]

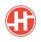	HealthifyMe	Tushar Vashisht	AI‐based calorie tracking, diet and workout plans, integration with fitness devices, and live coach support	[53]

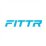	FITTR	Jitendra Chouksey	Community support, personalized diet and fitness plans, certified coaches, and transformation challenges	[54]

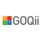	GOQii	Vishal Gondal	Fitness tracking, health coaching, integrated wearable devices, and rewards for healthy behaviors	[55]

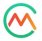	Carb Manager	Brian Davis	Low‐carb and keto meal tracking, macro tracking, recipes, and integration with fitness devices	[56]

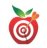	Cronometer	Aaron Davidson	Detailed nutrition tracking, barcode scanning, diet planning, and biometric tracking	[57]

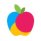	Yazio	Sebastian Weber	Calorie counting, meal planning, recipes, and diet tracking	[58]

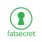	FatSecret	Lenny Moses	Calorie counter, diet calendar, weight tracker, and community support	[59]

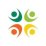	Baritastic	Dan Abeling	Designed for bariatric patients, food and weight tracking, reminders, and educational resources	[60]

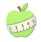	Calorie Counter	Sergey Oreshko	Calorie counting, diet planning, exercise logging, and diabetes tracking features	[61]

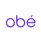	Obé Fitness	Ashley Mills, Mark Mullett	Live and on‐demand fitness classes, personalized workout plans, and community engagement	[62]

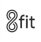	8fit	8fit Team	Workout routines, meal planning, goal tracking, and progress monitoring	[63]

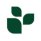	Lifesum	Fredrik Broman	Personalized meal plans, calorie counter, health tracking, integration with fitness devices	[64]

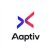	Aaptiv	Ethan Agarwal	Audio‐based workouts, personalized fitness plans, and expert trainers for different fitness levels	[65]

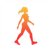	WalkFit	Welltech Apps Ltd.	Step tracking, walking challenges, personalized goals, integration with health trackers	[66]

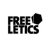	Freeletics	Freeletics Team	Personalized fitness plans, high‐intensity training, and bodyweight exercises for weight management	[67]

As summarized in Table 2, available applications span calorie‐ and nutrition‐tracking tools (e.g., MyFitnessPal, Lose It!, and Cronometer), behavior‐change platforms (e.g., Noom and WW), fitness‐centric apps (e.g., Fitbit and Freeletics), and tools tailored to specific populations (e.g., Baritastic and Carb Manager).

However, it is crucial to recognize that not all commercially available mobile applications qualify as DTx under regulatory definitions (e.g., FDA standards). True DTx is characterized by evidence‐based behavior change strategies that have been clinically validated through published peer‐reviewed research and are subject to regulatory oversight. While self‐monitoring tools and wearable devices provide valuable insights into behavioral patterns—such as energy expenditure, physical activity, and sleep—most function purely as general wellness tools rather than clinically validated therapeutic interventions.

The level of clinical evidence varies considerably among the applications listed in Table 2. Notably, platforms like Noom and WW (Weight Watchers) have undergone rigorous, peer‐reviewed clinical evaluation. Most recently, a 68‐week RCT (*n* = 427) demonstrated that Noom Weight participants achieved significantly greater weight loss than educational controls at both 16 weeks (−1.2% vs. +0.6%; *p* = 0.001) and 68 weeks (−4.1% vs. +1.5%; *p* < 0.001), with 41% of Noom participants achieving clinically significant weight loss of ≥ 5% at 68 weeks [44]. Importantly, the study also demonstrated that the program did not increase eating disorder risk. These findings build upon earlier observational data from 35,921 users showing a 77.9% weight reduction rate [45]. For WW, a 12‐month three‐arm RCT (*n* = 271; BMI 27–40) compared the Weight Watchers Online program alone (WWO), WWO combined with an ActiveLink activity tracker (WWO + AL), and an online newsletter control. At 3 months, WWO participants achieved significantly greater weight loss (2.7 kg [95% CI, 2.0–3.5]) than controls (1.3 kg [95% CI, 0.5–2.0]; *p* = 0.01), with a significantly higher proportion achieving ≥ 5% weight loss (24.5% vs. 9.4%; *p* = 0.01). At 12 months, although between‐group weight loss differences were no longer statistically significant, WWO participants maintained a significantly higher proportion of ≥ 5% weight loss compared to controls (25.5% vs. 12.9%; *p* = 0.04). Notably, the addition of an activity tracking device did not confer additional benefits over the online program alone [47].

Applications such as MyFitnessPal and Fitbit have been extensively studied as adjunctive tools in clinical trials, although their efficacy as standalone interventions remains inconsistent. A within‐subjects validation study demonstrated that MyFitnessPal estimates of energy intake were statistically equivalent to weighed food intake (mean percent error 3.7%) under semi‐controlled free‐living conditions, with high user preference (82%) compared to alternative augmented‐reality applications, supporting its accuracy as a self‐monitoring tool [39]. However, a 6‐month RCT (*n* = 212) conducted in primary care settings found that simply introducing MyFitnessPal to overweight patients did not produce significantly greater weight loss compared to usual care (mean between‐group difference, −0.30 kg; *p* = 0.63), suggesting that the app alone, without additional behavioral support, may be insufficient for meaningful weight reduction [40]. In contrast, a subsequent 12‐week RCT (*n* = 105) demonstrated that when MyFitnessPal was paired with tailored goals and behavioral components, participants achieved clinically significant weight loss of approximately 2.4 to 2.8 kg across all intervention arms, irrespective of self‐monitoring strategy [41]. Similarly, an 8‐week weight loss trial (*n* = 47) comparing the Lose It! smartphone app with a smartphone memo and traditional paper‐and‐pencil tracking found that app users recorded dietary data significantly more consistently than the paper group (*p* = 0.042); however, all groups achieved comparable weight loss with no significant between‐group differences, suggesting that while the app improves self‐monitoring adherence, it does not independently enhance weight loss outcomes [52]. Taken together, these findings suggest that commercial self‐monitoring apps can be effective for weight loss when integrated with structured behavioral support but are unlikely to produce significant results when used in isolation.

For Fitbit, Wang et al. conducted a 6‐week RCT (*n* = 67) among overweight and obese adults comparing Fitbit One alone versus Fitbit One combined with daily short message service (SMS)‐based physical activity prompts. While the Fitbit‐only group achieved a small but significant within‐group increase in moderate to vigorous intensity physical activity (MVPA) (+4.3 min/week; *p* = 0.04), the addition of SMS prompts produced significantly greater increases in steps and physical activity only during the first week of the intervention, with the effect diminishing thereafter. These findings suggest that wearable trackers can modestly increase physical activity but that simple SMS‐based prompts alone may be insufficient for sustained behavioral change [49]. Furthermore, Hartman et al. demonstrated that combining Fitbit with MyFitnessPal and phone‐based coaching in women with BMI ≥ 27.5 kg/m^2^ (*n* = 54) produced significantly greater weight loss over 6 months compared to usual care (4.4 kg vs. 0.8 kg; *p* = 0.004), with greater increases in MVPA significantly associated with greater weight loss (*p* = 0.01). These findings collectively suggest that wearable activity trackers are most effective when integrated with dietary self‐monitoring tools and structured behavioral support [50].

Conversely, many other applications listed in Table 2 function primarily as self‐monitoring tools without published clinical validation meeting the standards of the Digital Therapeutics Alliance (DTA) or regulatory bodies. Therefore, clinicians and researchers must differentiate between applications offering evidence‐based therapeutic components and those serving as general wellness tools when evaluating their suitability for clinical obesity management.

### 3.4. Application of AI in Obesity Management

The dual burden of malnutrition, encompassing both undernutrition and overnutrition (obesity), impacts populations globally. The World Obesity Federation projects that over 51% of the global population (more than 4 billion people) will live with overweight or obesity by 2035 [68]. Consequently, significant healthcare resources are increasingly allocated to managing obesity and its preventable comorbidities, which are leading causes of avoidable mortality [69, 70]. The modern digital era provides researchers and clinicians with unprecedented volumes and modalities of health data, offering novel opportunities for advancing healthcare through sophisticated data analytics [71]. Handling large‐scale, complex, and unstructured data—such as text, images, videos, and audio—requires advanced computational capabilities and analytical tools [72].

Consequently, the healthcare sector has increasingly adopted AI, particularly machine learning (ML) and deep learning (DL) algorithms [73]. AI applications range from predictive modeling for disease outbreaks to medical imaging and behavioral modification strategies [74, 75]. In the context of obesity, ML and DL techniques have proven highly beneficial in identifying meaningful clinical patterns and analyzing complex associations between various risk factors and weight‐related outcomes [76]. For instance, predictive ML models have been utilized to forecast the development of childhood obesity and analyze genetic and anthropometric traits [77]. Furthermore, random forest (RF) and conditional RF models have been employed to elucidate the links between neighborhood environments and the prevalence of obesity [78], while convolutional neural network (CNN)‐based DL approaches have successfully extracted built‐environment features from satellite imagery to assess their regional impact on obesity rates [79].

Comparative studies frequently highlight the efficacy of AI models over traditional statistical techniques. Many studies report that AI models outperform conventional methods in prediction accuracy on test data and account for a greater share of variance in obesity prevalence [80]. ML models demonstrate exceptional adaptability when handling diverse variables and massive datasets, producing robust, generalizable conclusions [81, 82]. However, some literature suggests comparable performance between ML algorithms and traditional statistical methods [83]. Furthermore, findings regarding the superiority of specific AI models remain context‐dependent. While some studies found logistic regressions to exhibit superior accuracy compared to decision trees (DTs), Naïve Bayes (NB), and DL [84, 85], others concluded that DL models, data mining techniques, and logistic regressions were equally effective, or that DL yielded higher classification accuracy [86, 87].

Despite these promising advancements, the application of AI and ML in obesity prediction is not without limitations. Model performance is highly dependent on the quality, size, and representativeness of the input data, leading to variability in outcomes across different populations [80–82]. While ML models excel in handling high‐dimensional and nonlinear data, their performance gains over traditional statistical approaches are often marginal when applied to structured clinical datasets with limited sample sizes [83]. In several studies, conventional models such as logistic regression have demonstrated comparable or even superior performance to complex AI models, with greater transparency and ease of implementation [84–87]. Moreover, issues related to model interpretability, reproducibility, and external validity present significant barriers to clinical adoption [80, 83]. Therefore, future research should focus not only on improving predictive accuracy but also on enhancing explainability, generalizability, and clinical utility of AI‐driven models, alongside robust validation and standardization, to ensure their reliable integration into clinical obesity management.

### 3.5. Cost‐Effectiveness of DTx Compared to Traditional Treatments

Several studies indicate that DTx presents a highly cost‐effective alternative to conventional obesity management strategies. For example, randomized controlled trials have demonstrated that digital interventions can significantly reduce overall healthcare visits and resource utilization while achieving clinical outcomes comparable to intensive face‐to‐face treatments. By reducing the need for frequent in‐person appointments, DTx eases the financial and logistical burden on patients and institutions while improving the efficiency and quality of care through automation, continuous monitoring, and remote support. Consequently, the reduced reliance on acute medical care leads to better long‐term health outcomes and a lower incidence of obesity‐related complications—such as cardiovascular disease and type 2 diabetes—ultimately generating substantial cost savings over the long run [88, 89].

In addition, the integration of AI and ML within DTx enables the development of highly individualized care plans, potentially resulting in sustained therapeutic efficacy at progressively lower costs over time (Figure 4). Crucially, DTx dramatically improves healthcare accessibility, particularly for patients who face geographic, temporal, or logistical barriers to regular clinical visits [35, 90]. The user‐friendly nature of digital platforms encourages high‐frequency engagement, allowing patients to seamlessly integrate therapeutic interactions into their daily routines and develop lasting habits—a level of consistent behavioral reinforcement that traditional in‐person sessions often cannot achieve [91, 92].

**FIGURE 4:**
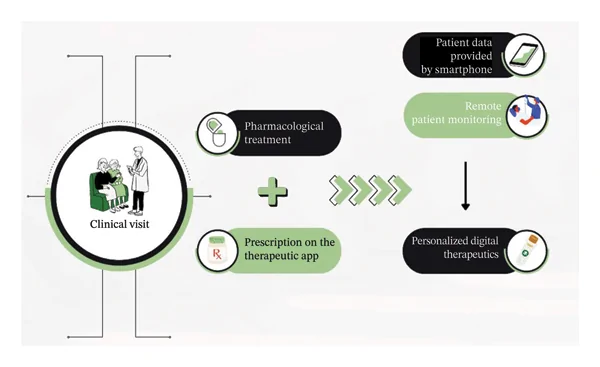
Conceptual framework for the integration of pharmacological therapy and digital therapeutics (DTx) in personalized obesity care.

## 4. Clinical Evidence and Trials of DTx

A new paradigm in personalized treatment, wherein tech‐centric interventions are tailored to individual patient needs, has emerged, largely driven by the growing success of DTx in chronic disease management, such as obesity and diabetes. However, these digital interventions must be supported by robust clinical data to ensure they provide measurable health benefits and serve as reliable components of comprehensive treatment plans. Clinical trials are essential to establish this evidence, providing a scientific foundation for the therapeutic claims of DTx applications [93]. Table 3 summarizes key clinical trials evaluating the efficacy of various DTx applications in obesity management.

**TABLE 3:** Clinical trials evaluating the digital therapeutics for the management of obesity.

Study objective	Key findings/results	Reference
Within‐subjects validation study assessing the accuracy of MyFitnessPal for estimating energy intake under semi‐controlled free‐living conditions	MyFitnessPal estimates were statistically equivalent to weighed food intake (mean percent error 3.7%), with high user preference (82%), supporting its accuracy as a self‐monitoring tool	[39]
6‐month RCT (*n* = 212) evaluating MyFitnessPal versus usual care in overweight primary‐care patients	Introducing MyFitnessPal did not produce significantly greater weight loss than usual care (−0.30 kg; *p* = 0.63); app usage declined sharply, with only ∼32% (34/105) still logging in by month 6	[40]
12‐week RCT (*n* = 105) evaluating MyFitnessPal paired with tailored goals and behavioral components	Participants achieved clinically significant weight loss of ∼2.4–2.8 kg across all intervention arms, irrespective of self‐monitoring strategy	[41]
68‐week RCT (*n* = 427) comparing the Noom Weight program with an educational control	Noom participants achieved significantly greater weight loss at 16 weeks (−1.2% vs. +0.6%; *p* = 0.001) and 68 weeks (−4.1% vs. +1.5%; *p* < 0.001); 41% achieved ≥ 5% weight loss at 68 weeks, with no increase in eating disorder risk	[44]
12‐month three‐arm RCT (*n* = 271; BMI 27–40) comparing Weight Watchers Online (WWO), WWO + ActiveLink tracker, and an online newsletter control	WWO produced greater weight loss than control at 3 months (2.7 vs. 1.3 kg; *p* = 0.01) and a higher proportion achieving ≥ 5% loss at 12 months (25.5% vs. 12.9%; *p* = 0.04); adding an activity tracker conferred no additional benefit	[47]
6‐week RCT (*n* = 67) comparing Fitbit One alone versus Fitbit One plus daily SMS physical‐activity prompts in overweight/obese adults	Fitbit alone produced a small significant within‐group increase in MVPA (+4.3 min/week; *p* = 0.04); added SMS prompts increased steps only during week 1, with the effect diminishing thereafter	[49]
6‐month RCT (*n* = 54) combining Fitbit, MyFitnessPal, and phone‐based coaching versus usual care in women with BMI ≥ 27.5 kg/m^2^	The combined intervention produced significantly greater weight loss than usual care (4.4 vs. 0.8 kg; *p* = 0.004); greater increases in MVPA were associated with greater weight loss (*p* = 0.01)	[50]
8‐week RCT (*n* = 47) comparing the Lose It! smartphone app, a smartphone memo, and paper‐and‐pencil dietary tracking	App users recorded dietary data significantly more consistently than the paper group (*p* = 0.042), but all groups achieved comparable weight loss with no significant between‐group differences	[52]
RCT with 110 overweight individuals assessing the impact of a mobile app on health indicators	Minimal weight loss (0.357 kg; *p* = 0.7) but a significant reduction in HbA1c (−0.095%; *p* = 0.046)	[94]
RCT with 61 overweight patients studying app usage over time	Greater reduction in hip circumference, blood pressure, and intake of saturated fat and sugar‐sweetened beverages	[95]
Evaluation of Reactivate app usability among youth	High usability ratings; users indicated they would use the Reactivate app to monitor their growth for motivation and goal setting	[96]
RCT with 96 overweight adolescents comparing TECH and standard interventions	Both groups experienced weight loss, with the TECH intervention group demonstrating slightly greater improvement	[97]

Although DTx demonstrates promising potential in obesity management, the available clinical evidence presents mixed and sometimes conflicting findings. As summarized in Table 3, several randomized controlled trials report improvements in behavioral outcomes, user engagement, and certain metabolic parameters; however, the magnitude of weight loss is often modest or statistically nonsignificant. For instance, while some studies observed reductions in anthropometric measures and dietary risk factors, others reported minimal changes in body weight despite improved glycemic indicators such as HbA1c. These inconsistencies may be attributed to variations in study design, duration of intervention, sample size, and heterogeneity in patient populations, as well as differences in the functionality and intensity of digital interventions. Furthermore, adherence and long‐term engagement remain critical challenges, with declining user participation over time potentially limiting sustained clinical benefits.

Importantly, several methodological and behavioral factors may explain why some trials failed to demonstrate significant weight loss outcomes. First, many control groups received standard lifestyle counseling or minimal digital support, which may approximate the activity level and behavioral support received by intervention arms, thereby narrowing between‐group differences. Second, relatively short intervention durations (often < 6 months) limit the ability to capture sustained weight loss, which typically requires long‐term behavioral adaptation. Third, intervention intensity and personalization vary widely across studies; low‐frequency engagement, lack of adaptive feedback, or absence of human coaching components may reduce effectiveness. In addition, high attrition and drop‐out rates—commonly observed in digital interventions—can substantially bias outcomes, compromise statistical power, and underestimate true efficacy.

Collectively, these findings underscore that the clinical success of DTx is highly dependent on sustained engagement, adequate intervention intensity, and rigorous trial design. Future studies should prioritize longer follow‐up periods, standardized outcome measures, and optimized engagement strategies to more accurately assess the true therapeutic potential of digital interventions in obesity management.

## 5. Regulatory Considerations for DTx

DTx comprises high‐quality software programs utilized as therapeutic interventions for the diagnosis, treatment, mitigation, and prevention of diseases or disorders, aiming to improve patient outcomes. DTx represents a rapidly growing sector within the healthcare industry, driven by software‐based solutions. Globally, the regulatory landscape for DTx is evolving. In India, regulations for DTx are currently under development. Recently, the Ministry of Health and Family Welfare (MoHFW) proposed the Draft Digital Information Security in Healthcare Act (DISHA Bill) to regulate digital health data. Currently, DTx in India is regulated as SaMD under the Medical Devices Framework, overseen by CDSCO.

In the United States, DTx is regulated by the FDA. These products are classified as medical devices and require premarket review to ensure safety and efficacy according to SaMD guidelines. Similarly, the European Union has established a defined regulatory framework under the Medical Device Regulation (MDR) and In Vitro Diagnostic Regulation (IVDR). The EMA evaluates these products to ensure high‐quality standards and clinical effectiveness. Furthermore, the EU’s General Data Protection Regulation (GDPR) mandates the strict handling of patient data. In Brazil, the National Health Surveillance Agency (ANVISA) oversees DTx under medical device regulations. Australia regulates DTx through the Therapeutic Goods Administration (TGA), imposing stringent guidelines to establish safety and efficacy. In Japan, DTx is regulated by the Pharmaceuticals and Medical Devices Agency (PMDA) and requires premarket approval (PMA) [98, 99]. The global regulatory frameworks for medical devices, including risk classification, approval processes, and post‐market requirements, are summarized in Table 4.

**TABLE 4:** Global regulatory frameworks for medical devices, risk classification, approval, and post‐market requirements.

Country	Regulatory body	Regulatory framework	Risk classification	Approval process	Post market requirements
USA	Food and Drug Administration (FDA)	FDA SaMD Guidelines; regulated under 21 CFR Part 820 Quality System Regulations	Based on patient risk: Class I (low) to Class III (high)	Premarket submissions (510(k), De Novo, or PMA) depending on risk	Post‐market surveillance, adverse event reporting, and software updates
Europe	European Medicines Agency (EMA) and National Authorities	EU Medical Device Regulation (MDR) (EU 2017/745); risk‐based classification	Classes I (low risk), IIa, IIb, and III (high risk)	CE marking after notified body review and conformity assessment	Vigilance reporting, periodic safety updates, and audits by notified bodies
Canada	Health Canada	Medical Devices Regulation (SOR/98‐282); aligned with IMDRF framework	Classes I (lowest risk) to IV (highest risk)	License application review and approval for Class II–IV device	Mandatory incident reporting and annual license renewals
India	Central Drugs Standard Control Organization (CDSCO)	Medical Devices Rules, 2017; SaMD treated as a medical device	Risk‐based: Class A (low) to Class D (high)	Requires approval for Class B‐D devices	Post‐market surveillance, periodic device audits, and recall systems
Australia	Therapeutic Goods Administration (TGA)	Therapeutic Goods Act 1989; IMDRF‐based classification system	Classes I (low), II (high), with additional SaMD guidance	Inclusion in the Australian Register of Therapeutic Goods (ARTG)	Mandatory incident reporting and post‐market audits
Japan	Pharmaceuticals and Medical Devices Agency (PMDA)	Pharmaceuticals and Medical Devices Act (PMD Act)	Classified as Class I, II, III, or IV based on patient risk	Premarket approval or certification by PMDA or a registered body	Post‐approval re‐certification and adverse event reporting

### 5.1. Regulatory Landscape of SaMD

The digital healthcare sector is undergoing a significant revolution, particularly concerning the development of SaMD with minimal human interference. These technologies are designed to facilitate diagnostic interventions, follow‐up treatment plans, symptomatic management, and disease surveillance. The widespread adoption of SaMD is driven by technological innovations, the expansion of telemedicine, and the increasing demand for remote patient monitoring. According to the International Medical Device Regulators Forum (IMDRF), SaMD is defined as software intended for one or more medical purposes that performs these purposes without being part of a hardware medical device. Its applications range broadly from clinical monitoring and diagnosis to active treatment and disease prevention [100].

A defining characteristic of SaMD is its platform independence, allowing integration with computers, smartphones, and standalone hardware. Global health crises, such as the COVID‐19 pandemic, significantly accelerated the adoption of digital health innovations. SaMD became a critical tool for remote patient oversight, providing safe clinical insights and treatment directions. Consequently, the global SaMD market was valued at approximately USD 1443.69 million in 2022 and continues to revolutionize the healthcare industry by enhancing accessibility and driving innovative patient care [101].

### 5.2. U.S. FDA Regulations for SaMD

The U.S. FDA regulates SaMD based on the level of risk it poses to patient safety and its clinical effectiveness. Aligning with IMDRF principles, the FDA assesses the functions of the SaMD rather than the platform it operates on. Key regulatory considerations include clinical validation, cybersecurity, real‐world performance monitoring, and risk‐based classification. The FDA’s Pre‐Certification (Pre‐Cert) Program aims to streamline approvals for developers demonstrating a culture of quality and organizational excellence. Concurrently, traditional regulatory pathways such as the 510(k) clearance, De Novo classification, and PMA are utilized depending on the established risk levels. Compliance with Quality System Regulations (QSR), Good Machine Learning Practices (GMLP) for AI‐based SaMD, and robust post‐market surveillance are mandatory. Developers must ensure transparency, continuous real‐world evidence collection, and ongoing risk management to sustain regulatory approval [102, 103].

Clinical trials are critical for establishing the safety, effectiveness, and stability of SaMD, particularly when supporting a 510(k) submission. Unlike conventional medical devices or pharmaceuticals, SaMD relies on software‐driven outputs, necessitating tailored clinical evaluation approaches. A rigorously designed clinical trial must incorporate formal hypotheses to drive statistical analysis, ensuring suitable evaluation of the device’s performance. These hypotheses—whether testing for superiority, noninferiority, or equivalence—dictate crucial study parameters such as sample size and analytical methodology. While statistical design principles for traditional devices are well‐established, guidelines for SaMD remain continuously evolving. Consequently, developers are required to employ robust methodologies aligned with current best practices, utilizing real‐world data and post‐market surveillance to scientifically substantiate regulatory submissions [104].

### 5.3. EU Regulations for SaMD

The European regulatory framework for SaMD shares fundamental similarities with U.S. regulations, aligning with criteria established for health IT and conventional medical devices. While the EU MDR does not explicitly define “SaMD,” it recognizes software as a specific category of medical device. Medical Device Software (MDSW) is defined as software intended to be used, alone or in combination, for a purpose specified in the definition of a medical device under the MDR or IVDR, regardless of whether it is independent or influences the use of hardware. The classification of SaMD under the EU MDR requires a rigorous determination of its risk profile, ranging from low to high risk.

Similar to other medical devices, SaMD must comply with the Quality Management System (QMS) provisions specified in Annex IX of the EU MDR. The IMDRF provides foundational guidance on implementing these QMS principles during SaMD development. The EU MDR formally recognizes and integrates IMDRF clarifications regarding the clinical evaluation of SaMD. Manufacturers must provide robust clinical data supporting the safety, performance, and analytical accuracy of their software interventions commensurate with their designated risk class. The general safety and performance requirements (GSPR) detailed in Annex I of the EU MDR apply universally to all medical devices, addressing critical aspects such as risk management, design validation, usability, and cybersecurity. Depending on the risk classification, SaMD may require a conformity assessment—often involving the evaluation of technical documentation by a Notified Body—prior to market entry. Thus, the EU MDR establishes an exhaustive regulatory framework featuring strict IMDRF‐aligned risk classification and rigorous QMS enforcement [105, 106].

### 5.4. Ethical Considerations and Health Equity

Despite their substantial clinical potential, DTx for obesity management raises significant ethical and health equity concerns. The continuous collection of sensitive personal and biometric data necessitates stringent protocols regarding data privacy, cybersecurity, informed consent, and transparent data usage policies. Furthermore, if AI‐driven diagnostic or treatment algorithms are trained on nonrepresentative datasets, algorithmic bias may emerge, thereby diminishing the efficacy of the DTx across diverse demographic groups.

From a health equity perspective, the widespread adoption of DTx among socioeconomically disadvantaged populations—particularly in low‐ and middle‐income countries—may be severely hindered. Barriers include restricted access to smartphones, lack of stable internet connectivity, low digital literacy, the absence of language‐appropriate content, and prohibitive costs associated with software subscriptions or wearable integrations. Therefore, to ensure the fair and ethical deployment of DTx in obesity treatment, stakeholders must prioritize inclusive design, affordability, cultural adaptability, and robust data governance frameworks [99].

## 6. Challenges, Research Gaps, and Future Perspectives

Despite the growing popularity of DTx in obesity management, several significant barriers hinder its widespread integration into routine clinical practice and healthcare facilities. A primary challenge is the scarcity of robust, long‐term longitudinal data evaluating sustained weight loss maintenance and durable behavioral changes after the initial intervention period concludes. Furthermore, sustaining high levels of patient adherence over extended periods remains a critical hurdle, as DTx platforms inherently require continuous user participation. This indicates that the long‐term efficacy of digital health technologies relies heavily on the development of advanced patient engagement strategies. Future platforms must incorporate hyper‐personalized feedback loops, sophisticated gamification, and effective behavioral reinforcement mechanisms to maintain user motivation. In addition, the seamless integration of digital health technologies into traditional healthcare systems remains highly limited. Enhancing the interoperability between DTx platforms and electronic health records (EHRs), alongside aligning them with existing clinical workflows, is essential. Such integration would equip healthcare professionals with comprehensive tools to remotely monitor the treatment progress of patients with obesity and effectively embed digital interventions into structured, multidisciplinary care plans.

Moving forward, future research must prioritize large‐scale, randomized controlled trials with extended follow‐up periods to validate long‐term clinical outcomes. There is also a pressing need to leverage AI to design more personalized treatment algorithms and to address the “digital divide” by ensuring that these technologies are accessible to populations of diverse socioeconomic backgrounds and digital literacy levels. Overcoming these clinical, technical, and systemic barriers is imperative for DTx to realize its full potential as a foundational component of sustainable, long‐term obesity management.

## 7. Conclusion

DTx is poised to play a transformative role in modern medicine. It is essential for healthcare professionals—including physicians, nurses, and allied health workers—to embrace these emerging technologies as valuable tools within clinical practice. Much like traditional diagnostic instruments, DTx offers evidence‐based, clinically validated interventions that can enhance diagnostic accuracy, personalize treatment, and significantly improve patient outcomes. When properly integrated, DTx can support clinicians in delivering tailored care, particularly in areas where conventional approaches may fall short—such as continuous behavioral modification and long‐term obesity management. Far from replacing the role of medical professionals, these tools serve to augment their capabilities, offering unprecedented precision and scalability in treatment. While some clinicians may initially express hesitation toward adopting DTx, it is imperative to recognize that medical science is constantly evolving, and clinical practice must evolve alongside it. As DTx becomes more widely implemented, healthcare providers will be expected to supervise their use, ensure clinical appropriateness, and adapt their workflows to maximize therapeutic benefits. Embracing DTx is not only a reflection of technological progress but also a necessary step toward more efficient, responsive, and patient‐centered care.

Based on the synthesized evidence, the following specific recommendations are proposed to advance the field of obesity management: For researchers, future randomized controlled trials evaluating DTx for obesity should incorporate a minimum follow‐up period of 12 months to accurately capture long‐term weight maintenance. Furthermore, studies should standardize outcome reporting using validated measures (e.g., BMI, waist circumference, and HbA1c) and ensure the inclusion of diverse demographic populations to improve the generalizability of findings. For clinicians, the DTA Evidence Standards Framework or equivalent regulatory classification criteria should be rigorously applied when evaluating applications for clinical use. Importantly, DTx should be integrated into structured obesity care pathways alongside traditional dietary and behavioral counseling, rather than being deployed in isolation. For policymakers, robust reimbursement frameworks for evidence‐validated DTx must be developed to incentivize adoption. In addition, equitable access initiatives—including subsidized digital literacy programs and affordable smart‐device schemes—are urgently needed to bridge the digital divide and ensure these vital therapies reach socioeconomically underserved populations.

## Author Contributions

Mayuri, Bidisha Bhattacharya, Kunal Kar, and Pravin Pawar: visualization and writing–original draft. Inderbir Singh: conceptualization and writing–review and editing. Tanikan Sangnim: writing–review and editing. Kampanart Huanbutta: supervision and writing–review and editing.

## Funding

No funding was received for this research.

## Conflicts of Interest

The authors declare no conflicts of interest.

## Data Availability

No new data were created or analyzed in this study. Data sharing is not applicable to this article.
